# Genomic Dissection of Growth Traits and Spatial Independence from Breed-Defining Loci in Jining Grey Goats

**DOI:** 10.3390/ani16162593

**Published:** 2026-08-19

**Authors:** Yongbo Chen, Tianxu Liu, Aowu Wu, Jingchao Cao, Di Lian, Zhengxing Lian

**Affiliations:** State Key Laboratory of Animal Biotech Breeding, Beijing Key Laboratory for Animal Genetic Improvement, College of Animal Science and Technology, China Agricultural University, Beijing 100193, China; s20253040853@cau.edu.cn (Y.C.); liutianx@cau.edu.cn (T.L.); bs20243040464@cau.edu.cn (A.W.); s20233040741@cau.edu.cn (J.C.)

**Keywords:** Jining Grey goat, low-depth whole-genome sequencing, growth traits, genome-wide association study, selective-sweep analysis, candidate marker panel

## Abstract

Improving growth while preserving breed-defining characteristics is a central challenge in indigenous livestock breeding. We addressed this issue in Jining Grey goats using low-depth whole-genome sequencing and a conservative multi-evidence marker-prioritization framework. The discovery cohort included 70 performance-graded goats at 3 months (*n =* 37) and 6 months (*n =* 33; mean depth, 2.07×), and 47 seven-day-old goats from the same project were used for exploratory early-life trend analysis (mean depth, 2.10×). After site-level filtering, phasing, and within-cohort imputation, masking-based assessment of 120,000 genotype cells yielded 94.38% exact genotype concordance and a hard-dosage correlation of 0.919. Performance grading captured consistent differences in body weight and body-size traits, but the small 3-month Conventional group required conservative interpretation. Selective-sweep analyses highlighted breed-background regions, including signals near *KIT*, whereas GWAS and superior-genotype comparisons prioritized candidate growth loci, including chr10:78518687 for six-month chest girth and literature-supported genes involved in muscle growth and developmental regulation. Overlap and permutation analyses indicated limited physical overlap between growth-prioritized loci and major selective-sweep regions. A 99-SNP candidate panel showed exploratory prediction potential (maximum cross-validated r ≈ 0.47), whereas seven-day analyses showed only nominal trends without FDR significance. These findings provide a preliminary marker resource for future Jining Grey goat breeding and conservation, pending external validation, denser genotyping, and functional confirmation.

## Simple Summary

The Jining Grey goat is a Chinese indigenous goat breed valued for its gray coat, high prolificacy, and adaptation to local production systems, but its growth performance still requires genetic improvement. This study used growth records and low-depth whole-genome sequencing from 70 performance-graded goats to search for candidate genomic regions associated with growth traits. We also examined 47 seven-day-old goats from the same project to explore whether prioritized markers showed early-life trait trends. To reduce reliance on any single analysis in a small population, we combined phenotype comparisons, population-structure analysis, selective-sweep scans, genome-wide association analysis, superior-genotype screening, positional annotation, and exploratory genomic prediction. The results identified a 99-SNP candidate marker panel and showed limited physical overlap between growth-associated candidate regions and major selective-sweep regions related to breed background. However, the findings should be interpreted as preliminary because the cohort was small, sequencing depth was low, imputation was performed within the cohort, and the seven-day analysis did not provide independent validation. Overall, this study provides a conservative candidate-marker resource for future validation and for balanced improvement of growth traits while conserving key characteristics of Jining Grey goats.

## 1. Introduction

Indigenous livestock breeds are reservoirs of genetic diversity shaped by long-term natural adaptation, local management, and artificial selection. In many local goat breeds, economically relevant traits are closely linked to breed identity, including coat color, reproductive performance, disease resilience, and adaptation to regional production systems. Genetic improvement in such populations therefore requires a balanced strategy: increasing growth and meat-production potential while avoiding unintended erosion of breed-specific characteristics and locally adapted genetic backgrounds [1,2].

The Jining Grey goat is a representative Chinese indigenous goat genetic resource [3,4]. It is valued for its grey coat, high prolificacy, adaptability to local farming conditions, and importance in regional goat production [5,6]. Previous descriptions of this breed and related Chinese local goat resources have mainly emphasized phenotypic characteristics, reproductive performance, coat-color features, conservation value, and production potential. However, genome-wide marker resources specifically designed for growth-trait improvement in Jining Grey goats remain limited. This gap restricts the development of marker-assisted strategies that could support early screening while preserving the genetic features that define the breed.

Low-depth whole-genome sequencing has become an attractive genotyping strategy for local livestock populations because it can provide genome-wide variant information at a lower cost than high-depth sequencing [7,8]. When combined with stringent variant filtering, phasing and genotype imputation, this approach can support population-structure analysis, selective-sweep scans and exploratory genome-wide association studies. Nevertheless, low-depth sequencing also introduces important uncertainty. Genotype errors, imperfect imputation, rare-variant instability, and small reference-free cohorts can affect downstream association and prediction analyses. For this reason, low-depth WGS studies require explicit quality control, imputation assessment, and cautious interpretation, especially when the sample size is modest.

Growth traits are complex quantitative traits influenced by many loci of small effect, population structure, and environmental variation [9,10]. In small or unbalanced cohorts, a single genome-wide association study may be underpowered and may generate false-positive signals if relatedness, allele-frequency differences, imputation uncertainty, or group imbalance are not adequately considered [11,12]. Therefore, candidate-marker discovery in local breeds benefits from a multi-evidence framework in which GWAS signals are interpreted together with phenotypic differentiation, selective-sweep evidence, superior-genotype contrasts, positional annotation, prior biological knowledge, and exploratory validation in additional samples [13,14]. Such integration does not replace independent validation, but it can help prioritize robust candidate regions for future testing.

A further challenge is to distinguish loci potentially related to growth improvement from genomic regions associated with breed background. Selective-sweep regions enriched for breed-defining characteristics, such as coat color, reproductive traits, or local adaptation, may overlap with growth-associated candidates or lie in nearby linkage-disequilibrium regions. Extensive overlap would require careful assessment before marker-assisted breeding, whereas limited physical overlap would provide preliminary genomic evidence that growth-related markers may be explored without directly targeting major breed-background regions. However, such conclusions should be based on coordinate-level comparisons, sensitivity analyses, and appropriate null expectations, rather than on a single arbitrary overlap window.

In this study, we analyzed low-depth whole-genome sequencing and growth-related phenotypes in Jining Grey goats to develop an exploratory candidate-marker resource for growth traits. The study integrated phenotypic differentiation, population-structure assessment, selective-sweep analysis, GWAS, superior-genotype screening, candidate-marker prioritization, exploratory genomic prediction, and within-project early-life marker–trait trend analysis. The objectives were to (i) characterize growth differentiation between performance groups; (ii) identify candidate loci supported by GWAS and complementary evidence lines; (iii) evaluate the extent of physical overlap between growth-related candidate regions and breed-background selective-sweep regions; and (iv) construct a preliminary SNP panel for subsequent validation in larger, independent populations.

## 2. Materials and Methods

### 2.1. Study Design, Animal Sources, and Ethical Approval

This study was designed as an exploratory candidate-marker discovery analysis in Jining Grey goats. The analytical workflow combined phenotypic record curation, performance-grade grouping, low-depth whole-genome sequencing (WGS), variant calling, phasing and within-cohort imputation, population-structure analysis, selective-sweep scans, genome-wide association studies (GWAS), multi-evidence marker prioritization, exploratory genomic estimated breeding value (GEBV) assessment, and seven-day early-life trend analysis.

The present analysis used existing animal samples, phenotypic records, and sequencing data generated within the project framework. Animals were sampled at Shanxian Jining Grey Goat Industry Research Institute, Shandong Province, China. Project records indicated that 293 Jining Grey goats were sampled between December 2024 and November 2025, with concurrent recording of body weight, coat color, sex, body height, oblique body length, chest girth, reproductive performance, litter size, lactation performance, and kid growth information. Among these animals, 117 blood samples were submitted to the National Facility for Phenotypic and Genetic Research of Model Animals from May to July 2025 for DNBSEQ-T7 whole-genome resequencing.

From the sequenced animals, 70 goats with usable growth phenotypes and performance-grade information were retained as the discovery cohort. This cohort comprised 37 animals measured at 3 months and 33 animals measured at 6 months. The 3- and 6-month datasets contained no overlapping individual identifiers and were therefore treated as age-specific cross-sectional cohorts rather than repeated measurements of the same animals. An additional 47 seven-day-old animals from the same sequencing batch were used for within-project exploratory early-life marker–trait analysis.

Animal management was conducted under the routine standardized management system of the source farm. Because complete daily feed formulations, nutrient-composition records, and environmental-control measurements were not available in the project records, these variables were not modeled as explicit covariates. This limitation was considered when interpreting phenotype comparisons, GWAS, and prediction analyses.

The use of existing animal samples, phenotypic records, and sequencing data in this study was approved by the Laboratory Animal Welfare and Animal Experimental Ethics Committee of China Agricultural University (protocol code Aw31706202-1-06; date of approval: 13 July 2026).

### 2.2. Phenotypic Records, Performance Grading, and Derived Traits

Phenotypic records collected at 7 days, 30 days, 3 months, and 6 months were organized and harmonized by sample identifier. Recorded traits included body weight (BW), body height (BH), body length (BL), chest girth (CG), back height or related body-size indices (BG), chest width (CW), front width or related body-size indices (FW), cannon circumference or related limb indices (TG), and head length (HL, when available).

Performance grades in the discovery cohort were taken from project sampling records and farm grading information. Special-grade goats were assigned to the Superior group, whereas first-grade and below-first-grade animals were combined as the Conventional group. At 3 months, the discovery cohort included 34 Superior and 3 Conventional animals. At 6 months, it included 24 Superior and 9 Conventional animals. Because the 3-month Conventional group was small, effect-size estimates and nominal *p*-values from this comparison were interpreted cautiously and were not used as stand-alone evidence for marker selection.

Age-specific growth and body-conformation traits were analyzed separately. The 3-month analyses included BW3, BH3, BL3, CG3, ADG_2_3, BMI3 and CGI3; the 6-month analyses included BW6, BH6, BL6, CG6, ADG_5_6, BMI6 and CGI6. Average daily gain (ADG), body mass index (BMI) and chest girth index (CGI) were calculated from the recorded body-weight and body-size measurements according to the phenotype-processing table. Continuous-trait differences between the Superior and Conventional groups were assessed using Welch’s *t*-test when applicable, and Cohen’s d was used to summarize effect size.

### 2.3. Low-Depth Whole-Genome Sequencing Data Processing and Variant Quality Control

Sequencing data for the discovery cohort and the seven-day exploratory cohort were generated in the same DNBSEQ-T7 sequencing batch. Sequencing depth was estimated from raw FASTQ base counts divided by the ARS1 reference-genome length (2,922,813,246 bp). The discovery cohort had a mean estimated depth of 2.07× (range, 1.06–2.55×), and the seven-day cohort had a mean estimated depth of 2.10× (range, 1.48–2.63×). These values confirmed that the study was based on low-depth WGS and that genotype uncertainty needed to be handled through stringent filtering and imputation assessment.

FASTQ integrity and sample matching were checked before downstream processing. Read processing, alignment, and variant calling were performed with nf-core/sarek v3.8.1 [15,16]. The pipeline included fastp-based read quality control [17], alignment to the goat ARS1 reference genome (Capra_hircus.ARS1.dna.toplevel.fa) using BWA-MEM2 [18,19], duplicate marking, CRAM/BAM processing, and GATK HaplotypeCaller gVCF generation. The workflow was run in a containerized environment with explicit reference FASTA, FASTA index, sequence dictionary, and BWA-MEM2 index files. Detailed library-construction metrics such as insert-size distribution and per-library PCR-cycle information were not available in the project records and were therefore not used as filtering covariates.

### 2.4. Variant Calling, Filtering, Phasing, and Imputation Assessment

Joint variant processing was restricted to autosomes 1–29. Variants were normalized using bcftools norm and filtered to retain PASS, biallelic single-nucleotide polymorphisms with minor allele count greater than or equal to 2. SNPs were excluded if they met any of the following GATK hard-filtering criteria: QD < 2.0, FS > 60.0, SOR > 3.0, MQ < 40.0, MQRankSum < −12.5, or ReadPosRankSum < −8.0. Before downstream analysis, variants were additionally filtered for missingness, allele frequency, and Hardy–Weinberg equilibrium using PLINK [20,21]. The global genotype-resource filter used --geno 0.05, --mind 0.10, --maf 0.01, and --hwe 1 × 10^−6^ midp keep-fewhet.

Population-structure analyses used a relaxed sample-missingness threshold (--mind 0.40) to retain comparator samples for genetic-background visualization. For age-specific GWAS datasets, monomorphic variants were removed, and cohort-level PLINK filtering used --maf 0.05, --geno 0.10, and --hwe 1× 10^−6^ midp keep-fewhet. After these filters, the retained GWAS matrices contained 265,073 SNPs for the 3-month cohort and 430,673 SNPs for the 6-month cohort.

Low-depth genotypes were phased and imputed with Beagle v5.5 (beagle.27Feb25.75f.jar) [22,23]. No external reference panel was used; imputation was performed within the study cohort. The final phased VCF defined DR2 and DS fields in the header, but these fields were not populated in the retained records. Therefore, Beagle DR2 was not used as a post-imputation filtering criterion, and dosage-based accuracy statistics from Beagle DS could not be reported from the final retained VCF.

To evaluate imputation reliability, we added an internal masking-based validation analysis using high-confidence observed genotypes from the pre-phasing filtered VCF. Chromosomes 1, 10, and 23 were selected, and 40,000 genotype cells per chromosome were masked, yielding 120,000 masked genotype cells in total. The masked data were re-imputed using the same Beagle settings as in the main analysis. Accuracy was summarized as exact hard-genotype concordance, hard-dosage correlation, concordance by genotype category, and concordance by minor-allele-frequency class. Because downstream GWAS used imputed hard genotypes, fixed post-imputation genotype-level DP and GQ filters were not applied to the final imputed dataset; instead, genotype reliability was evaluated through this masking framework and through cohort-level site filters.

### 2.5. Population-Structure Analysis

Population structure was evaluated to describe the genetic background of the Jining Grey goat study cohort relative to comparator goat populations and to support interpretation of selective-sweep analyses. Autosomal SNPs were pruned for linkage disequilibrium before principal component analysis (PCA) and ancestry-component estimation. PCA was performed on the pruned genotype matrix, and the first three principal components were visualized. ADMIXTURE v1.3.0 was run across K = 2 to K = 8, and the optimal number of ancestral components was evaluated using cross-validation error [24,25]. These analyses were used for genetic-background characterization rather than for assigning causal status to any candidate locus.

### 2.6. Selective-Sweep Scans and Line a Integration

Selective-sweep evidence was summarized using a window-based Line A framework. The genome was scanned using 50 kb windows with a 25 kb step. The final integrated Line A table included F_ST_ and XP-EHH evidence [26,27], XP-CLR and iHS evidence [28,29], and the nucleotide–diversity ratio [30]. F_ST_, π ratio, XP-CLR, and XP-EHH were calculated for Jining Grey goats compared with Bezoar, Boer, Chengdu Brown, and Jintang Black comparator groups, whereas iHS was calculated within the Jining Grey goat population.

For each statistic and contrast, values were standardized as z-scores within the corresponding statistic/contrast column. Directionality was set so that higher standardized values represented stronger differentiation or stronger evidence of recent selection in the Jining Grey goat lineage. For each window, the composite score was calculated as the mean of all available standardized values, and the number of non-missing supporting statistic/contrast columns was recorded as the support count. Runs of homozygosity and Tajima’s D were considered during exploratory auditing but were not included in the final Line A composite table used for candidate-marker prioritization. The resulting selective-sweep regions were interpreted as breed-background or population-differentiation context rather than direct evidence of growth-trait causality.

### 2.7. Genome-Wide Association Studies and Line B Definition

GWAS were performed separately in the 3- and 6-month cross-sectional cohorts using GEMMA v0.98.5 mixed linear models. A centered relatedness matrix was used as the kinship matrix to account for genetic relatedness among animals. Because the 3- and 6-month datasets represented separate age-specific cohorts, age was not fitted as a within-model covariate. All animals were sampled from the same source farm and processed within the same sequencing project; detailed management and library-batch covariates were not available for stable model adjustment. Given the small cohort sizes, additional covariate-rich models were not used as the primary analysis to avoid unstable parameter estimation. This modeling choice was treated as a limitation of the study.

Traits analyzed at 3 months included BW3, BH3, BL3, CG3, ADG_2_3, BMI3 and CGI3. Traits analyzed at 6 months included BW6, BH6, BL6, CG6, ADG_5_6, BMI6 and CGI6. Manhattan plots were generated for each age-trait combination. *p* < 5 × 10^−8^ was shown as a stringent genome-wide reference threshold, and *p* < 1 × 10^−5^ was used as a suggestive threshold for exploratory candidate-locus prioritization. These thresholds were used to guide marker discovery and were not interpreted as proof of causality.

To obtain relatively independent association signals for Line B, PLINK clumping was applied using P1 = 1 × 10^−4^, P2 = 1 × 10^−3^, r^2^ = 0.2, and a 500 kb window [21,31]. The resulting clumped index variants and their nearest positional annotations were used as GWAS-supported candidate signals. GWAS summary statistics for the retained cohort-specific GWAS datasets are provided as Supplementary Data.

### 2.8. Superior-Genotype Screening, Prior-Gene/QTL Annotation, and 99-SNP Panel Construction

The candidate-marker panel was constructed by integrating three lines of evidence. Line A represented selective-sweep regions from the window-based composite analysis. Line B represented GWAS clumped index variants. Line C represented variants showing allele-frequency differences between the Superior and Conventional groups. Prior-gene and QTL information were used only as contextual support and were not treated as independent proof of functional causality.

For superior-genotype screening, allele frequencies were compared between the Superior and Conventional groups within the discovery cohort. Candidate variants were retained when they showed evidence of enrichment in the Superior group and passed cohort-level allele-frequency and missingness filters. To reduce redundancy, variants were grouped by genomic proximity, including local support around candidate loci and 500 kb bin-level pruning where applicable. Markers with stronger statistical evidence, broader evidence-line support, and acceptable minor allele frequency were prioritized. The final 99-SNP panel consisted of candidate markers supported by at least one of the three evidence lines, with integrated evidence scores ranging from 1 to 3.

Candidate-gene annotation was performed as positional annotation. For each marker, the nearest gene, distance to the nearest gene, and variant context were recorded as intragenic, upstream, downstream, or intergenic using a 100 kb annotation window. AnimalQTLdb overlap was evaluated as positional co-localization [32]. Nearest-gene labels and QTL overlaps were used to guide interpretation and prioritization, but they were not considered equivalent to functional validation or causal-gene evidence.

### 2.9. Limited-Overlap Analysis, Sensitivity Analysis, and Local LD Context

To evaluate whether growth-candidate regions and breed-background selective-sweep regions showed extensive coordinate overlap, Line A selective-sweep windows were compared with physical intervals around Line B GWAS clumped index variants. Intervals of ±100 kb, ±250 kb, and ±500 kb around Line B index variants were generated. Coordinate overlap between Line A windows and Line B intervals was calculated by chromosome and summarized as the number of overlapping regions, total overlap length, and overlap proportion [33].

Because coordinate overlap alone cannot establish genomic independence, chromosome-matched permutation analyses were performed. In each permutation, Line B intervals were randomly repositioned within the same chromosomes while preserving interval size and chromosome distribution. Observed overlap was then compared with the empirical null distribution to estimate whether overlap was lower than expected by chance. Local linkage disequilibrium around Line B index variants was also summarized within ±500 kb windows, including maximum r^2^ values and counts of neighboring variants exceeding r^2^ thresholds of 0.2, 0.5, and 0.8. The results were interpreted as limited physical overlap and local LD context, not as proof of complete genomic independence.

### 2.10. Exploratory GEBV Analysis

Exploratory GEBV analysis was performed to assess whether the retained marker set captured prediction-relevant genetic signal in the discovery cohort. Genotype matrices were generated from the filtered and imputed SNP data, and prediction models were evaluated for age-specific growth traits. Models included BayesB and a GBLUP-equivalent ridge-regression framework [34,35] implemented in R v4.5.2 using the BGLR package v1.1.4.

Predictive performance was estimated using five-fold cross-validation. In each fold, the model was trained in the training subset and evaluated in the held-out subset. Prediction accuracy was summarized as the Pearson correlation between observed phenotypes and predicted values across folds, with mean accuracy, standard deviation, and standard error reported. Because of the limited sample size, these analyses were interpreted as exploratory benchmarks rather than production-ready genomic selection models.

### 2.11. Seven-Day Exploratory Marker–Trait Analysis

The 47 seven-day-old animals were used for within-project exploratory early-life marker–trait analysis. Candidate loci from the 99-SNP panel were defined in a BED file and recalled from CRAM files using bcftools mpileup and bcftools call. For each candidate SNP, genotype, depth, and allele-support information were extracted when available.

Eight seven-day phenotypes were evaluated: BW7d, BH7d, BL7d, CG7d, BG7d, CW7d, FW7d and TG7d. Marker-trait combinations were considered testable only when call rate was at least 0.8, the number of tested animals was at least 35, seven-day minor allele frequency was at least 0.03, and at least two dosage classes were present. Association trends were summarized using allele-dosage-based effect direction and rank-based correlation statistics. Multiple testing was controlled with the Benjamini–Hochberg false discovery rate [36]. Nominal *p* < 0.05 results were described as exploratory early-life trends only and were not interpreted as independent confirmatory evidence.

### 2.12. Statistical Software, Reporting Standards, and Reproducibility

Phenotypic statistics, effect-size calculations, coordinate-overlap analysis, permutation testing, marker–trait trend analysis, and visualization were performed using R, Python 3.14, PLINK 1.9, bcftools 1.22, bedtools 2.17.0, GEMMA 0.98.5, Beagle 4.0, and related open-source packages. Unless otherwise stated, *p*-values are reported as nominal values, and false-discovery-rate-adjusted results are explicitly indicated. Significance symbols in figures denote nominal thresholds only: ns, *p* ≥ 0.05; *, *p* < 0.05; **, *p* < 0.01; and ***, *p* < 0.001.

All candidate loci, annotation classes, overlap summaries, imputation-validation summaries, GEBV cross-validation summaries, and retained cohort-specific GWAS summary statistics were organized into Supplementary Tables or Supplementary Data Files to support reviewer inspection and reproducibility.

## 3. Results

### 3.1. Cohort Structure, Low-Depth Sequencing, and Phenotypic Differentiation

The discovery cohort comprised 70 Jining Grey goats with matched growth phenotypes and low-depth whole-genome sequencing data. It included 37 animals sampled at 3 months of age and 33 animals sampled at 6 months of age. These two age groups represented age-specific cross-sectional cohorts rather than repeated measurements of the same individuals. An additional 47 seven-day-old animals from the same project were retained for exploratory early-life marker–trait assessment.

**Figure 1:**
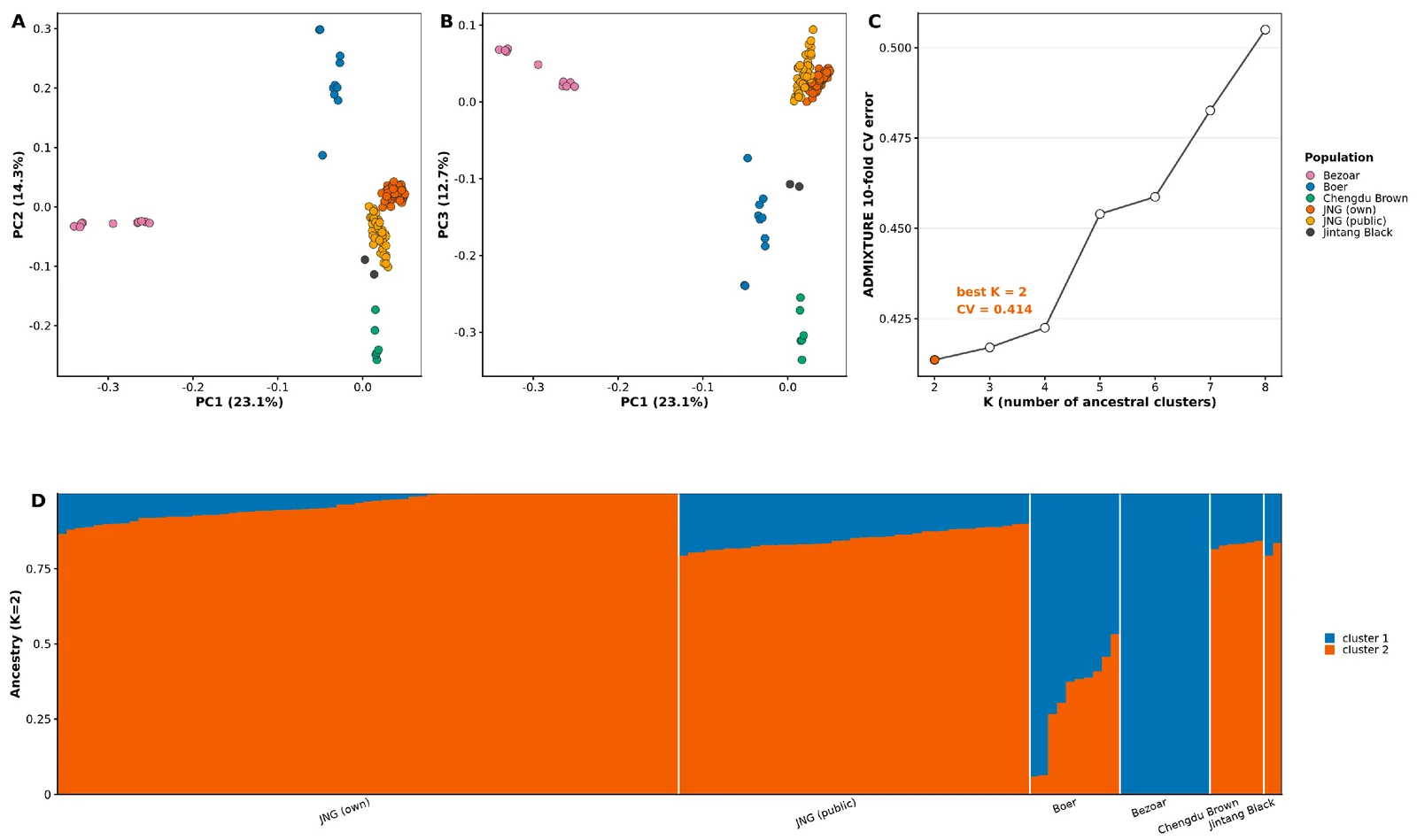
Population structure of Jining Grey goats and comparator populations. (**A**,**B**) Principal component analysis. (**C**) ADMIXTURE 10-fold cross-validation error from K = 2 to K = 8. (**D**) Individual ancestry proportions at K = 2. JNG, Jining Grey goat.

Sequencing-depth estimates based on raw FASTQ base counts confirmed the low-depth nature of the dataset. The discovery cohort had a mean depth of 2.07× (range, 1.06–2.55×), and the seven-day cohort had a mean depth of 2.10× (range, 1.48–2.63×). Population-structure analysis separated the Jining Grey goat study cohort from comparator populations, supporting the interpretation of selective-sweep signals in the context of breed background rather than treating all signals as growth-specific effects. The corresponding PCA and ADMIXTURE results are shown in Figure 1.

**Figure 2:**
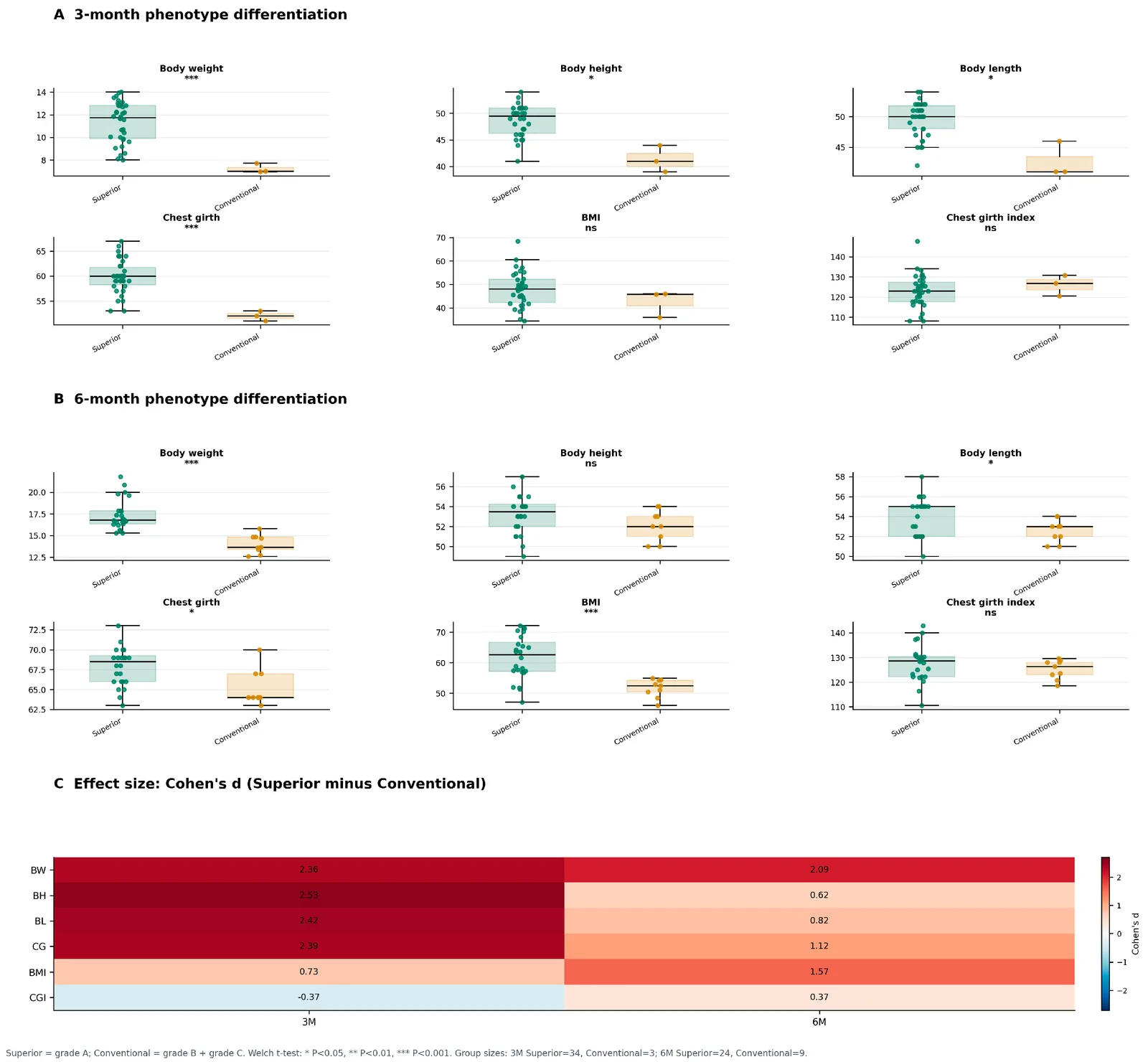
Phenotypic differentiation between Superior and Conventional Jining Grey goats. (**A**) Body-weight and body-size traits at 3 months of age. (**B**) Corresponding traits at 6 months of age. (**C**) Effect-size heatmap showing Cohen’s d for Superior minus Conventional animals. Group comparisons used Welch’s *t*-test. Because the 3-month Conventional group contained only three animals, the 3-month results were interpreted as descriptive evidence of phenotypic differentiation rather than as definitive group-level inference.

Performance grading captured clear but unevenly sampled phenotypic contrasts. At 3 months, the Superior group (*n =* 34) showed higher body weight, body height, body length, and chest girth than the Conventional group (*n =* 3). At 6 months, the Superior group (*n =* 24) maintained favorable trends relative to the Conventional group (*n =* 9), particularly for body weight, body length, and chest girth. Because the 3-month Conventional group contained only three animals, the 3-month significance tests and Cohen’s d estimates were interpreted cautiously. The main inference from this analysis was therefore based on the consistency of effect direction and the recurrence of growth advantages at 6 months, rather than on *p*-values alone. The phenotypic comparisons are summarized in Figure 2.

### 3.2. Imputation Assessment Supported Population-Level Use of the Retained Hard Genotypes

Low-depth sequencing required careful evaluation of genotype reliability before downstream analysis. The final phased VCF defined DR2 and DS fields in the header, but these fields were not populated in the retained records; consequently, Beagle DR2 was not used as a post-imputation filtering criterion. To evaluate the reliability of the retained hard genotypes, we performed a masking-based assessment using high-confidence observed genotypes from chromosomes 1, 10, and 23.

Across the three chromosomes, 120,000 high-confidence genotype cells were masked and re-imputed. All masked cells were recovered as non-missing genotypes. The weighted exact genotype concordance was 94.38%, and the weighted hard-dosage correlation was 0.919. Concordance was highest for reference homozygotes (98.21%) and lower for heterozygous and non-reference genotypes (83.89% and 85.97%, respectively). These results indicate that the imputed hard genotypes were suitable for population-level screening and candidate-locus prioritization, while also showing that heterozygous and non-reference genotypes remain more error-prone in an approximately 2× sequencing dataset.

Because dosage fields were not retained, downstream analyses were interpreted using hard-genotype results rather than Beagle dosage-based association statistics. This limitation was considered when interpreting GWAS signals, superior-genotype contrasts, exploratory genomic prediction, and seven-day marker–trait trends.

### 3.3. Selective-Sweep Integration Highlighted Breed-Background Candidate Regions

Selective-sweep evidence was summarized using a window-based Line A integration framework. The final integrated analysis combined F_ST_, π ratio, XP-CLR, iHS, and XP-EHH evidence across 50 kb windows with a 25 kb step. Rather than assigning functional causality to individual genes, this analysis was used to identify genomic regions likely to reflect breed background and historical selection in Jining Grey goats.

**Figure 3:**
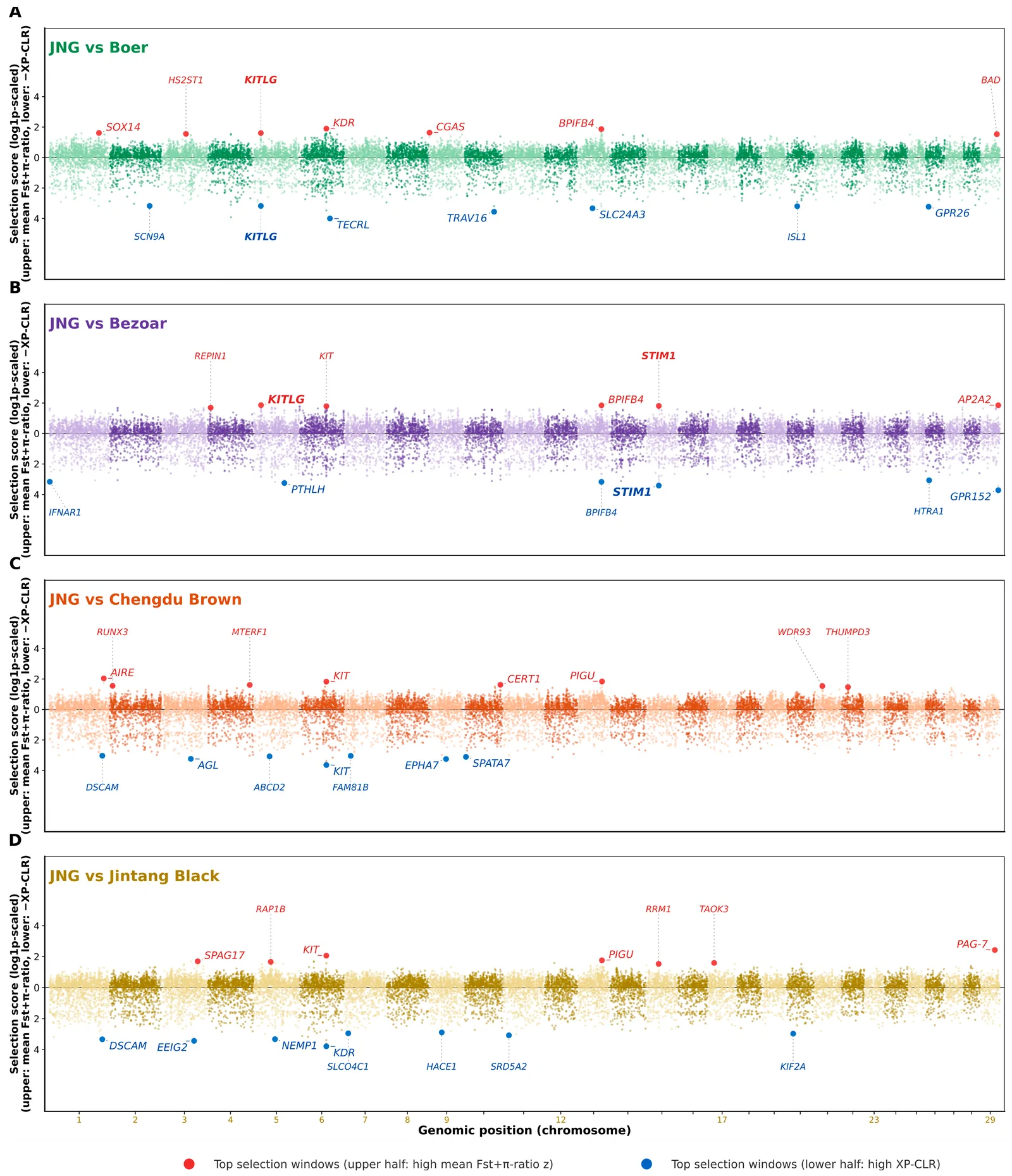
Genome-wide selective-sweep landscape of Jining Grey goats compared with reference goat populations. Signals are shown for Jining Grey goats compared with Boer, Bezoar, Chengdu Brown, and Jintang Black goats. The upper half shows windows with high standardized mean F_ST_ and π ratio signals, and the lower half shows windows with high XP-CLR signals. Labeled genes indicate positional candidates near top-ranked windows and were used as contextual rather than causal evidence.

The strongest integrated signals were concentrated on chromosome 6, where several high-ranking windows occurred in or near the *KIT* region. The top window reached a composite z-score of 12.05 and had broad support across the available statistics. Additional high-ranking windows were located near genes such as *NEMP1*, *ISL1*, *TECRL*, *GPR152*, *MED23*, *STIM1*, *CERT1,* and *SRD5A2*. These loci were interpreted as positional candidates within selective-sweep regions, with the recurrent chromosome 6 signal providing genomic context for breed-characteristic traits rather than direct evidence of growth regulation. The top selective-sweep regions and the genome-wide selective-sweep landscape are summarized in Table 1 and Figure 3, respectively.

### 3.4. GWAS Identified Candidate Association Signals for Growth-Related Traits

Genome-wide association analyses were performed for seven traits or derived indices at 3 months and seven traits or derived indices at 6 months using GEMMA mixed linear models. After final filtering and cleaning, the retained cohort-specific GWAS summary files contained 30,220 to 30,221 SNP rows for the 3-month analyses and 38,076 to 38,078 SNP rows for the 6-month analyses. These summary statistics are provided as Supplementary Data for reproducibility.

The strongest association was detected for 6-month chest girth at chr10:78518687:C:T (*p* = 6.86 × 10^−8^; β = 3.77). This locus had complete genotype availability in the 6-month cohort (33/33 animals), a minor allele frequency of 0.333, and genotype counts of 0/0 = 1, 0/1 = 20, and 1/1 = 12. Variant-level quality metrics were consistent with retained PASS status, and leave-one-out analysis supported the stability of the signal. Nevertheless, given the small 6-month sample size, this SNP was interpreted as a strong candidate association signal rather than a causal variant.

**Figure 4:**
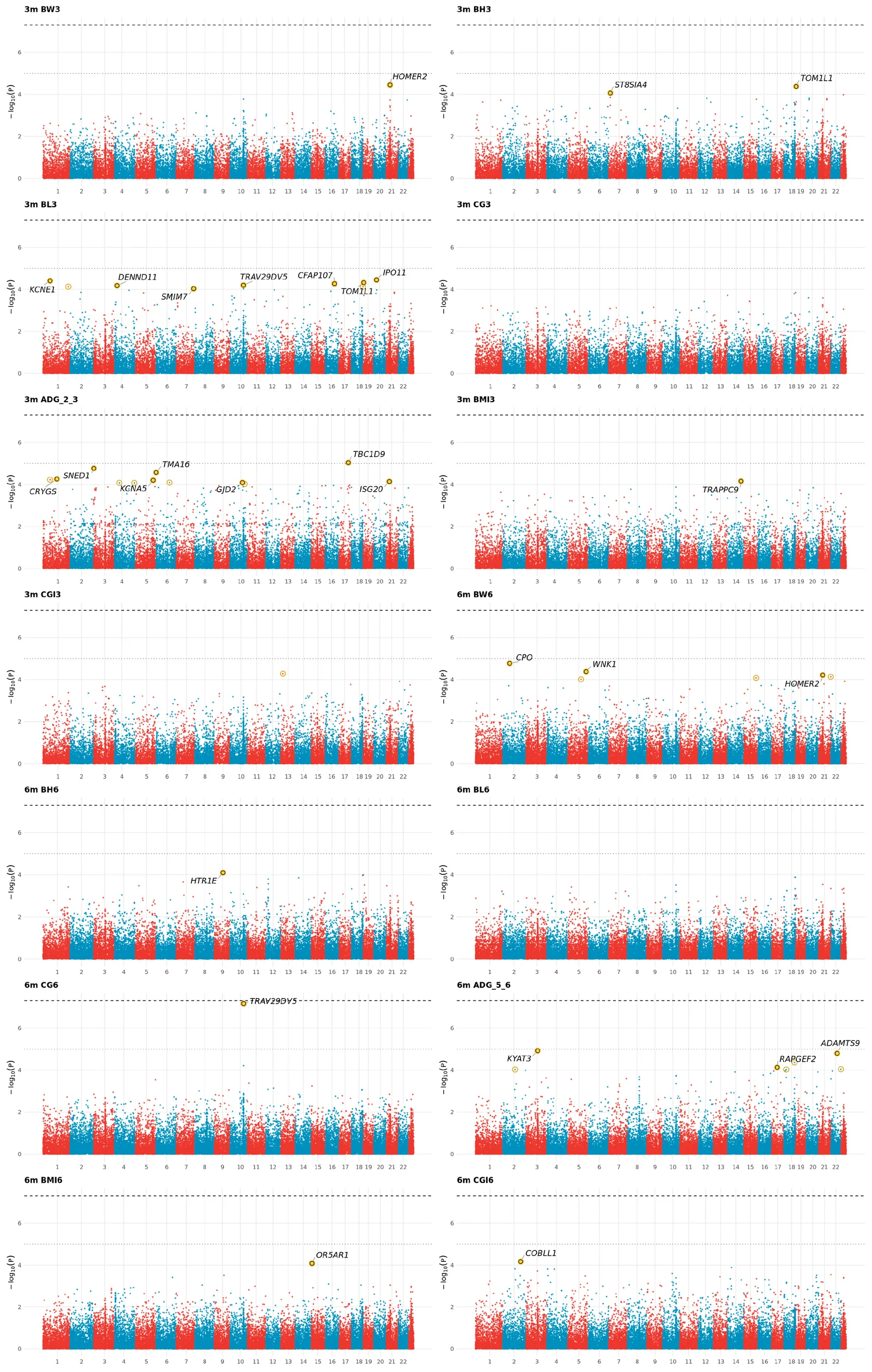
GWAS Manhattan plots for growth-related traits in Jining Grey goats. Each panel represents a GEMMA mixed-linear-model analysis for one age-trait combination. Highlighted variants indicate clumped index SNPs retained for candidate-locus prioritization. Dashed and dotted horizontal lines indicate the cohort-specific Bonferroni and 1/N suggestive thresholds used for visualization. Because retained SNP counts differed slightly among traits, the approximate Bonferroni thresholds were *p* = 1.65 × 10^−6^ for the 3-month analyses and *p* = 1.31 × 10^−6^ for the 6-month analyses, and the corresponding 1/N suggestive thresholds were *p* = 3.31 × 10^−5^ and *p* = 2.63 × 10^−5^, respectively. Because the discovery cohorts were small (3 months, *n =* 37; 6 months, *n =* 33), the highlighted loci were interpreted as candidate association signals rather than definitive causal loci.

Additional candidate signals were observed for average daily gain, body weight, body length, and body height. Nearest-gene labels in the Manhattan plots were used only to summarize positional context. They were not treated as functional validation or as proof that the nearest gene mediated the associated phenotype. The complete retained cohort-specific GWAS summary statistics and the clumped index variants are provided in the Supplementary Files. The corresponding Manhattan plots and representative clumped index variants are shown in Figure 4 and Table 2, respectively.

### 3.5. Multi-Evidence Prioritization Defined a 99-SNP Exploratory Candidate-Marker Panel

Candidate markers were prioritized by integrating three lines of evidence: Line A selective-sweep evidence, Line B GWAS clumped index variants, and Line C Superior-group genotype contrasts. Prior gene and QTL information were used as contextual support, not as evidence of direct causality. This strategy produced a 99-SNP candidate-marker panel. The panel included 31 SNPs derived from Line B GWAS evidence and 68 SNPs derived from Line C superior-genotype evidence; 69, 32, and 71 markers had Line A, Line B, and Line C support, respectively, because individual markers could receive support from more than one evidence category.

The integrated marker scores ranged from 1.0 to 3.0, and the marker minor-allele frequencies ranged from 0.051471 to 0.477941, with a median of 0.114754. Positional annotation showed that 41 markers were intragenic, 21 were upstream within 100 kb, 26 were downstream within 100 kb, and 11 were intergenic. The median distance to the nearest annotated gene was 2485 bp. Exact SNP-level overlap with AnimalQTLdb entries was not observed in the final panel; therefore, QTL information was used only as regional and biological context.

**Figure 5:**
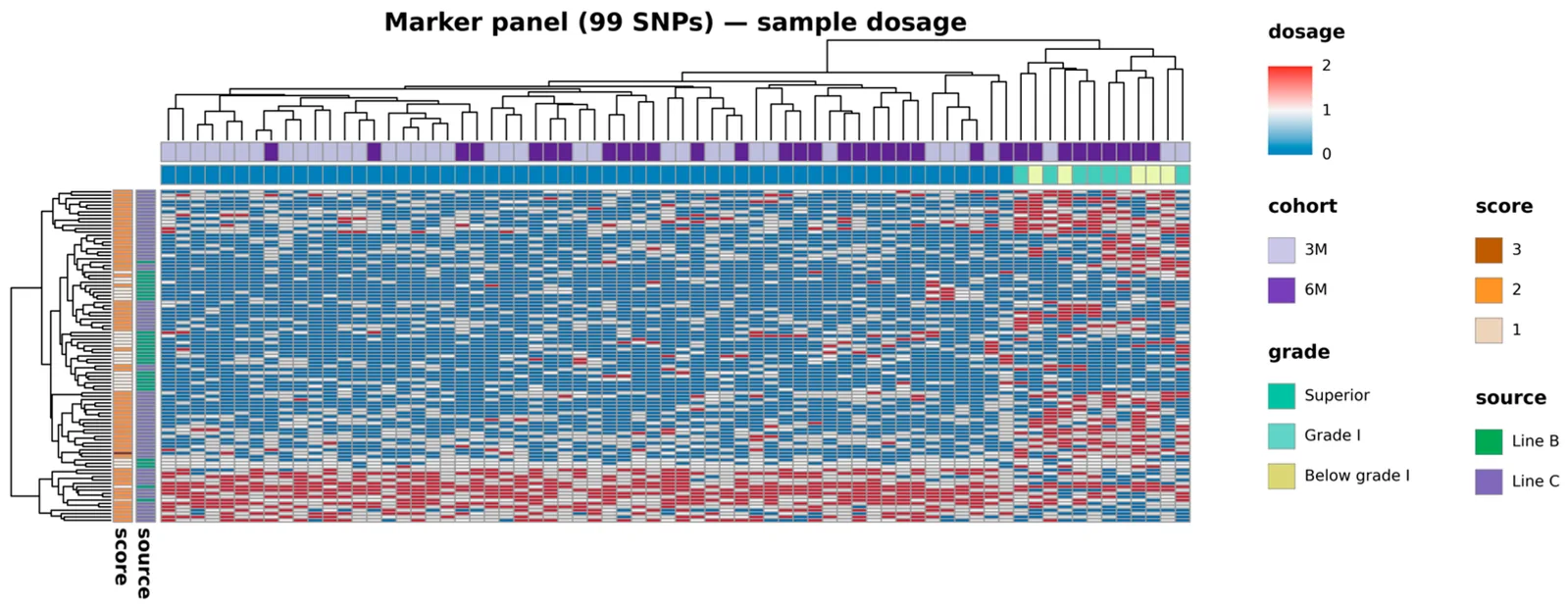
Multi-evidence 99-SNP candidate-marker panel. The heatmap summarizes evidence from selective-sweep regions, GWAS clumped index variants, Superior-group genotype contrasts, prior-gene context, and positional annotation. Tile annotations indicate the presence or absence of support from the corresponding evidence categories. Integrated scores range from 1.0 to 3.0, with higher values indicating broader multi-evidence support rather than a larger experimentally validated effect size. The panel is presented as an exploratory candidate-marker resource and not as a validated breeding assay.

The final panel contained markers near or within genes and pathways previously linked to muscle development, body size, growth regulation, and developmental biology, including *MSTN*, *KITLG*, *BMPR1B*, *ESR1*, *BMP2*, *BMP4,* and the *IGF1*/*IGF2* growth-regulatory axis. However, the evidence sources were not equivalent. Some candidates were supported by GWAS, whereas others were supported mainly by superior-genotype contrasts, prior-gene information, or positional annotation. Accordingly, these genes were described as candidate or literature-supported loci rather than experimentally validated regulators of growth. Prior-gene-supported contextual variants and complete positional annotations are provided in Supplementary Table S6a,b. The integrated 99-SNP candidate-marker panel is summarized in Figure 5.

### 3.6. Growth-Associated Candidates Showed Limited Physical Overlap with Breed-Background Selective-Sweep Regions

We next evaluated whether growth-associated candidates and breed-background selective-sweep regions showed extensive coordinate overlap. Because a fixed ±500 kb interval around GWAS index SNPs is an exploratory physical neighborhood rather than a true LD block for every locus, the overlap analysis was repeated at ±100 kb, ±250 kb, and ±500 kb and complemented by genome-matched permutation tests.

At ±100 kb, 11 Line A windows overlapped Line B neighborhoods, corresponding to 0.21% of all Line A windows and 15.56% of Line B index regions. At ±250 kb and ±500 kb, the observed overlaps were 23 and 46 Line A windows, respectively. Genome-matched permutation analysis showed that the observed overlap was not significantly lower than expected at ±100 kb (lower-tail *p* = 0.1156), but was lower than expected at ±250 kb (*p* = 0.0371) and ±500 kb (*p* = 0.0156). Local LD assessment around Line B index SNPs further showed that 31 of 45 index variants had a nearby marker with r^2^ ≥ 0.5 and 17 had a nearby marker with r^2^ ≥ 0.8 within ±500 kb.

Together, these analyses support limited physical overlap between the prioritized growth-candidate neighborhoods and the major breed-background selective-sweep windows. They do not establish complete genomic independence or rule out long-range regulatory effects. The results therefore provide preliminary coordinate-level evidence that future growth-improvement efforts may be designed alongside, rather than necessarily in conflict with, conservation of major breed-background regions.

### 3.7. Exploratory Genomic Prediction Suggested Limited but Detectable Predictive Potential

The 99-SNP panel was evaluated as an exploratory genomic prediction resource using five-fold cross-validation across 14 age-specific trait combinations. Prediction accuracy was measured as the Pearson correlation between observed phenotypes and predicted genomic values. This analysis was intended to benchmark marker-panel potential within the discovery dataset, not to demonstrate field-ready breeding performance.

**Figure 6:**
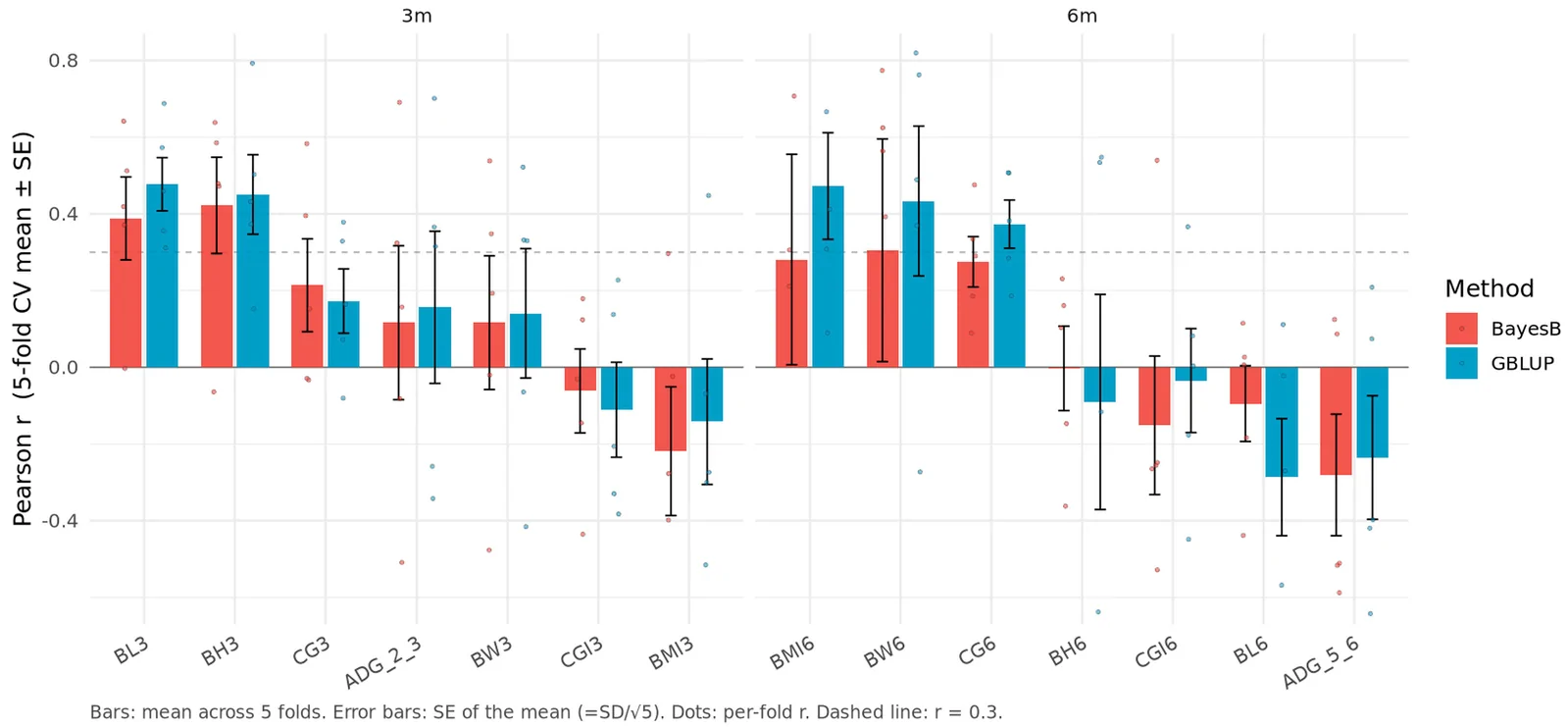
Exploratory genomic prediction performance of the 99-SNP candidate-marker panel. Prediction accuracy was evaluated by five-fold cross-validation and summarized as Pearson’s correlation between observed and predicted phenotypes. For each age-trait combination, individuals were partitioned into five folds within the corresponding cohort; each fold was used once as the test set and the remaining folds were used for model training. No external cohort or family-blocked validation split was used. Results indicate within-cohort predictive potential rather than immediate applicability for breeding deployment.

The highest mean cross-validation accuracies were observed for BL3 using the GBLUP-equivalent model (mean r = 0.477, SE = 0.069), BMI6 (mean r = 0.473, SE = 0.139), BH3 (mean r = 0.450, SE = 0.104), BW6 (mean r = 0.434, SE = 0.195) and CG6 (mean r = 0.373, SE = 0.063). These values indicate that the panel captured part of the growth-related variation in the discovery cohort. However, the small sample size and the limited number of animals per cross-validation fold make the estimates sensitive to sampling variation. The cross-validation results are summarized in Figure 6.

### 3.8. Seven-Day Samples Provided Exploratory Early-Life Marker–Trait Trends Without External Confirmation

Finally, the candidate-marker panel was examined in 47 seven-day-old animals from the same project. The seven-day analysis included eight early-life traits and 99 candidate markers. Of 792 possible marker–trait combinations, 592 passed testability criteria based on call rate, sample number, minor allele frequency, and dosage-class representation.

Twenty-four marker–trait combinations involving nine candidate markers reached nominal significance at *p* < 0.05. This number was lower than the 29.6 nominally significant results expected under the null hypothesis for 592 tests. No marker–trait combination remained significant after Benjamini–Hochberg false-discovery-rate correction, and the smallest nominal *p*-value was 6.31 × 10^−4^, with a corresponding FDR value of 0.32.

**Figure 7:**
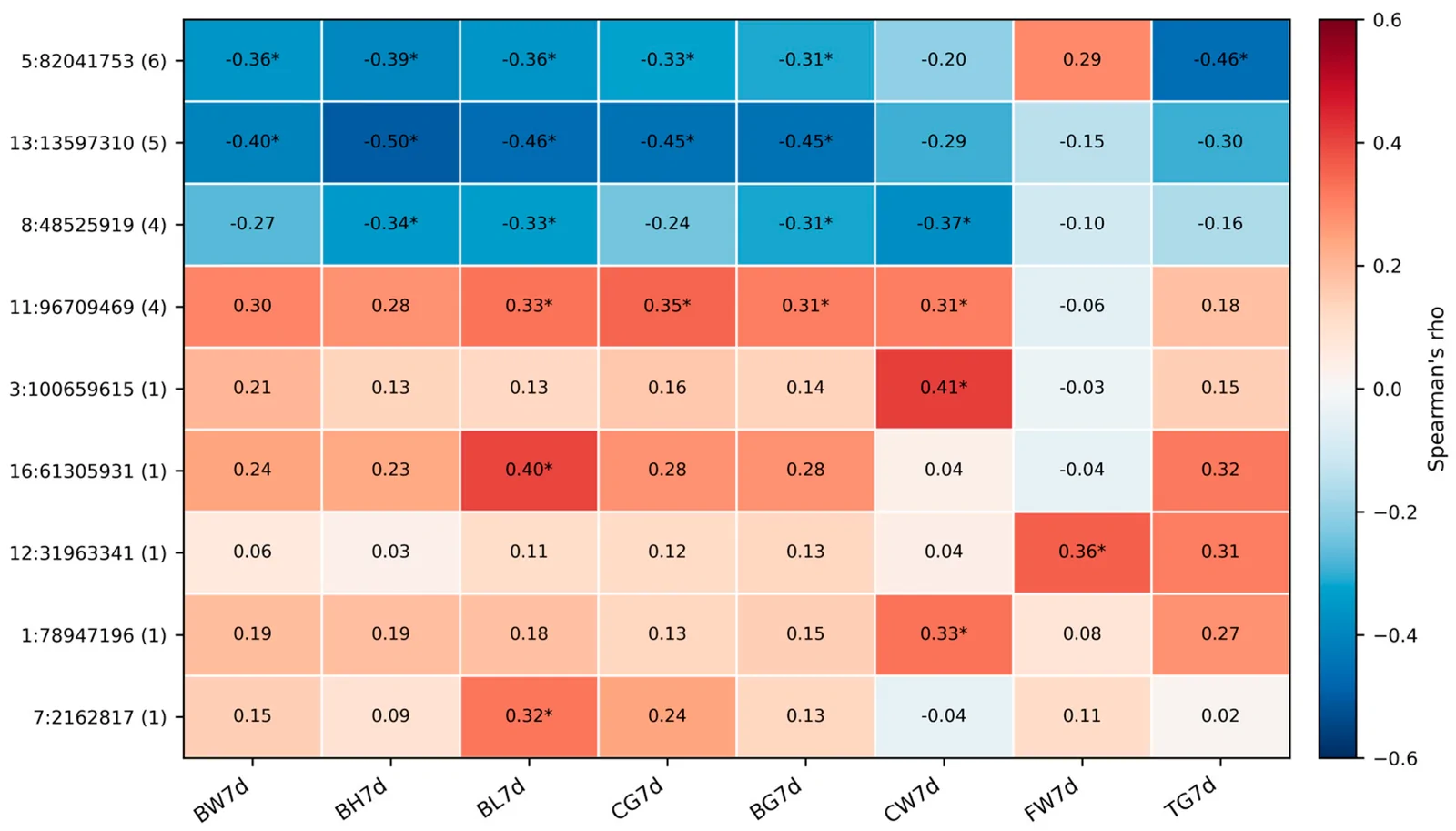
Seven-day exploratory marker–trait trend heatmap for prioritized candidate markers. Rows represent candidate markers from the 99-SNP panel that showed at least one nominal association with seven-day phenotypes. Numbers in parentheses indicate the number of seven-day traits with nominal *p* < 0.05. Cell values show Spearman’s rho, and asterisks denote nominal *p* < 0.05. No marker–trait combination remained significant after Benjamini–Hochberg FDR correction; therefore, these results were interpreted as within-project exploratory early-life trends rather than confirmatory evidence from an external validation cohort.

Therefore, the seven-day results were interpreted as exploratory early-life trends rather than confirmatory evidence from an external cohort. They provide hypotheses for future external validation in larger, independently sampled cohorts with deeper sequencing or orthogonal genotyping assays. The nine markers showing at least one nominal seven-day marker–trait trend are summarized in Supplementary Table S7b, and the complete marker–trait test results are provided in Supplementary Table S7a. The corresponding heatmap is shown in Figure 7.

## 4. Discussion

This study used the Jining Grey goat as a local-breed model to assess whether growth-related candidate loci could be prioritized while distinguishing them from major breed-background selective-sweep regions. Our findings support three main points. First, performance grading captured measurable differences in body weight and body size, particularly at 6 months of age. Second, low-depth WGS, after stringent filtering and masking-based imputation assessment, provided a practical genomic resource for exploratory candidate discovery. Third, the integrated evidence framework distinguished GWAS-supported loci, selective-sweep regions, superior-genotype signals, and prior-gene annotations, thereby reducing reliance on any single evidence type in a modest-sized cohort.

The use of low-depth WGS is relevant for indigenous livestock populations, where large-scale high-coverage sequencing is often constrained by cost and sample availability. Similarly to other livestock genomic studies using low-depth sequencing or imputation-based genotyping, this approach is most useful for population-level screening and candidate-marker development rather than for definitive individual-genotype inference [37,38]. In the present study, the mean sequencing depth was approximately 2×, which required conservative variant filtering and phasing/imputation before downstream analyses. The masking-based assessment partially addresses this limitation: across 120,000 masked high-confidence genotype cells, the weighted exact genotype concordance was 94.38%, and the hard-dosage correlation was 0.919. However, concordance was lower for heterozygous and non-reference genotypes than for reference homozygotes. Thus, the imputed genotypes support exploratory genomic screening, but key loci should still be confirmed by targeted genotyping before biological interpretation or breeding application.

The phenotypic comparison also requires conservative interpretation. The Superior group showed consistent advantages for several growth traits, and the pattern was more stable at 6 months. However, the 3-month Conventional group contained only three animals, which makes nominal *p*-values and effect-size estimates sensitive to sampling variation. For this reason, the phenotypic results should be viewed as evidence that the project grading records reflected broad growth-performance differences, not as precise estimates of all age-specific group effects. This limitation was considered in the downstream interpretation by emphasizing repeated directionality, effect-size patterns, and integration with genomic evidence rather than isolated 3-month significance tests.

For association mapping, the principal challenge was the small discovery cohort. The 3- and 6-month GWAS analyses included 37 and 33 individuals, respectively, which limits statistical power and increases the risk that individual peaks may be unstable [11,39]. We therefore treated GWAS results as candidate association signals and did not infer causality from single-locus significance. The strongest retained signal, chr10:78518687 for 6-month chest girth, was examined separately and remained a candidate locus rather than a confirmed causal variant. More generally, the marker-prioritization strategy was designed to reduce false-positive emphasis by combining GWAS clumped index variants, selective-sweep evidence, superior-genotype differences, positional annotation, and prior biological knowledge. This approach improves interpretability, but it does not remove the need for replication in larger cohorts.

A key strength of our interpretative framework is the separation of evidence categories for candidate genes. Nearest-gene annotation was used only as positional context and was not treated as functional proof. Some loci were supported directly by GWAS clumping, whereas others were supported mainly by superior-genotype differences, prior-gene lists, or QTL co-localization. Accordingly, genes such as *MSTN*, *IGF1*, *IGF2*, *BMP2* and *BMP4* are best interpreted as literature-supported or positional candidates within growth-related regulatory axes, rather than as direct GWAS-discovered causal genes in this dataset. This distinction is important because positional co-location cannot establish regulatory mechanisms, gene expression changes, or causality [40,41].

The biological interpretation is nevertheless coherent with known growth and breed-background processes in goats and other livestock. *MSTN* is a well-established regulator of skeletal-muscle development, whereas the IGF axis is central to growth, metabolism and postnatal body-size regulation [42,43]. *BMP2*, *BMP4* and *BMPR1B* belong to the TGF-β/BMP signaling framework, which connects skeletal development, tissue growth and reproductive biology [44,45]. In parallel, selection-supported regions involving *KIT* and *KITLG* are more plausibly related to breed-background traits such as coat color and developmental characteristics than to direct growth improvement [46,47]. *ESR1* and *BMPR1B* also provide plausible links to reproductive or developmental components of breed background [48,49]. These gene-level interpretations should therefore be read as hypotheses for follow-up studies rather than mechanistic conclusions from the current dataset.

We carefully evaluated the comparison between growth-candidate regions and breed-background selective-sweep regions to ensure conservative interpretation. The data support limited physical overlap, not complete genomic independence in a strict population-genetic sense. Sensitivity analyses across ±100 kb, ±250 kb and ±500 kb windows showed that observed overlap between Line A selective-sweep windows and Line B GWAS candidate neighborhoods was generally limited. Genome-matched permutation tests further indicated that overlap was lower than random expectation at the ±250 kb and ±500 kb scales, whereas the ±100 kb comparison was not significant. Together, these analyses provide preliminary coordinate-level evidence that major growth-candidate neighborhoods and major breed-background selective-sweep regions are not strongly co-localized in this dataset. They do not, however, exclude long-range linkage, polygenic correlations, or pleiotropic effects, all of which require larger pedigree or population datasets for formal evaluation.

The exploratory GEBV results should be interpreted in the same conservative framework. Five-fold cross-validation produced moderate prediction correlations for several traits, with the best values near r = 0.47. These results suggest that the retained genotype data and candidate markers capture some growth-related signal. However, the discovery cohort is too small to support deployment of a prediction model [50,51] and the 99-SNP panel should not be presented as a ready-to-use breeding tool. Its appropriate role is as a preliminary candidate-marker resource for technical genotyping, independent validation, and subsequent model recalibration in larger Jining Grey goat populations [52].

The seven-day analysis provides additional within-project screening information but should not be described as independent validation. The 47 seven-day-old samples were derived from the same project and sequencing batch. After applying testability filters, 592 marker–trait combinations were evaluated; 24 reached nominal *p* < 0.05, fewer than the 29.6 expected under the null, and none survived FDR correction. These findings therefore indicate exploratory early-life trends rather than validated marker–trait associations. Their value lies in identifying markers that may be worth testing in larger early-life cohorts with independent sampling, standardized phenotyping, and technical genotyping.

Overall, this study provides a structured candidate-discovery framework for a Chinese indigenous goat breed, but several limitations define the scope of inference. The discovery sample size was limited; the 3-month Conventional subgroup was unbalanced; genotype inference relied on approximately 2× sequencing and within-cohort imputation; the seven-day analysis was not externally independent; and the study lacked transcriptomic, functional, or gene-editing evidence. Future work should therefore prioritize larger cross-year and cross-farm cohorts, higher-confidence genotyping of the 99 candidate markers, independent evaluation of the chr10:78518687 signal, expression analysis in growth-relevant tissues, and formal testing of genetic correlations between growth traits and breed-background traits. Until such evidence is available, the present results should be considered preliminary genomic evidence for balanced breeding research in Jining Grey goats, rather than a validated marker-assisted selection system.

## 5. Conclusions

This study integrated low-depth whole-genome sequencing, phenotype-based performance classification, selective-sweep analysis, genome-wide association testing, candidate-marker prioritization, exploratory genomic prediction, and within-project seven-day trend analysis in Jining Grey goats. Our analyses identified growth-related candidate signals and breed-background selective-sweep regions that showed limited physical overlap across multiple window sizes and chromosome-matched permutation tests, rather than evidence for genomic independence. A 99-SNP candidate-marker panel was assembled from GWAS, selective-sweep, performance-grade differentiation, and prior-gene/QTL evidence, but this panel should be viewed as a preliminary resource for candidate prioritization and future validation.

Because the discovery cohort was small, the 3-month Conventional group was highly unbalanced, genotypes were derived from within-cohort imputation of low-depth sequencing data, and the seven-day analysis did not constitute independent validation, the current findings are not sufficient to recommend immediate marker-assisted selection or genomic selection. Instead, they provide preliminary genomic evidence and a hypothesis-generating framework for balanced breeding in Jining Grey goats. Larger cross-year and cross-farm cohorts, higher-confidence genotyping, expression or functional assays, and independent validation are required before practical breeding implementation.

## Figures and Tables

**Table 1:** Top consensus selective-sweep regions in Jining Grey goats.

Rank	Genomic Region	Composite z-Score	Support Score	Nearest Gene
1	chr6:70925001-70975001	12.05	12	Intergenic
2	chr6:70900001-70950001	11.89	12	Intergenic
3	chr6:70850001-70900001	7.23	12	*KIT*
4	chr6:70825001-70875001	6.038	12	*KIT*
5	chr5:55675001-55725001	5.728	12	*NEMP1*
6	chr13:61875001-61925001	5.629	10	Intergenic
7	chr20:27725001-27775001	5.061	12	*ISL1*
8	chr6:80475001-80525001	5.04	12	*TECRL*
9	chr29:46275001-46325001	4.897	12	*GPR152*
10	chr6:70875001-70925001	4.72	12	*KIT*
11	chr9:56825001-56875001	4.703	11	*MED23*
12	chr13:61850001-61900001	4.433	11	Intergenic
13	chr4:61450001-61500001	4.407	12	Intergenic
14	chr15:32225001-32275001	4.292	11	*STIM1*
15	chr10:94400001-94450001	4.191	12	*CERT1*
16	chr10:94375001-94425001	4.177	11	*CERT1*
17	chr1:108750001-108800001	4.072	12	Intergenic
18	chr11:14150001-14200001	4.062	12	*SRD5A2*
19	chr1:120900001-120950001	4.055	12	Intergenic
20	chr13:61900001-61950001	4.055	10	Intergenic

Note: The complete supporting information for selective-sweep regions and marker prioritization is provided in the Supplementary Materials.

**Table 2:** Representative GWAS clumped index variants for growth-related traits.

Locus	Index Variant	*p* Value	Trait	Cohort	Nearest Gene	Prior-Gene Support
chr10:78518687	10:78518687:C:T	6.86 × 10^−8^	CG6	6 m	Intergenic	N/A
chr17:54041252	17:54041252:C:A	9.14 × 10^−6^	ADG_2_3	3 m	*TBC1D9*	N/A
chr3:66306067	3:66306067:T:C	1.21 × 10^−5^	ADG_5_6	6 m	*KYAT3*	N/A
chr22:36885832	22:36885832:G:A	1.61 × 10^−5^	ADG_5_6	6 m	*ADAMTS9*	N/A
chr2:40697439	2:40697439:A:G	1.69 × 10^−5^	BW6	6 m	*CPO*	N/A
chr3:823629	3:823629:A:G	1.71 × 10^−5^	ADG_2_3	3 m	*SNED1*	N/A
chr6:2331351	6:2331351:T:C	2.67 × 10^−5^	ADG_2_3	3 m	*TMA16*	N/A
chr20:16397598	20:16397598:T:G	3.53 × 10^−5^	BL3	3 m	Intergenic	N/A
chr21:22793106	21:22793106:T:C	3.56 × 10^−5^	BW3	3 m	Intergenic	N/A
chr1:41327275	1:41327275:T:A	3.91 × 10^−5^	BL3	3 m	*KCNE1*	N/A
chr5:106623571	5:106623571:A:G	4.17 × 10^−5^	BW6	6 m	*RAD52*	N/A
chr19:4703195	19:4703195:A:G	4.22 × 10^−5^	BH3	3 m	*TOM1L1*	N/A
chr18:60790649	18:60790649:A:C	4.35 × 10^−5^	ADG_5_6	6 m	Intergenic	N/A
chr19:4703195	19:4703195:A:G	4.75 × 10^−5^	BL3	3 m	*TOM1L1*	N/A
chr13:13597310	13:13597310:G:A	5.26 × 10^−5^	CGI3	3 m	Intergenic	N/A

Note: The complete clumped-index list is provided in Supplementary Table S3.

## Data Availability

The raw sequencing data reported in this study have been deposited in the Genome Sequence Archive (GSA) at the National Genomics Data Center (NGDC), China National Center for Bioinformation, under accession number CRA047541. The corresponding dataset is available at: https://ngdc.cncb.ac.cn/gsa/browse/CRA047541 (accessed on 13 August 2026).

## Supplementary Materials

The following supporting information can be downloaded at: https://www.mdpi.com/article/10.3390/ani16162593/s1, Supplementary Tables.xlsx, including README, Table_Index, Supplementary Table S1a: per-sample sequencing coverage estimates; Supplementary Table S1b: sample metadata for the sequenced cohort; Supplementary Table S1c–e: phenotypic records for the 3-month, 6-month, and seven-day cohorts, respectively; Supplementary Table S2a: variant calling, filtering, and genotype quality-control summary; Supplementary Table S2b,c: masking-based imputation validation by chromosome and MAF class, and overall weighted imputation-validation summary, respectively; Supplementary Table S3: Line B GWAS clumped index variants; Supplementary Table S4: construction and evidence support of the 99-SNP candidate-marker panel; Supplementary Table S5: overlap-window sensitivity and chromosome-matched permutation results; Supplementary Table S6a: positional annotation of the 99-SNP panel, including nearest-gene distance and positional class; Supplementary Table S6b: prior-gene-supported contextual candidate variants; Supplementary Table S7a: complete seven-day marker–trait test results; Supplementary Table S7b: seven-day exploratory nominal marker–trait summary; Supplementary Table S8: five-fold GEBV cross-validation summary; and Supplementary Table S9: audit of the chr10:78518687 lead SNP. Supplementary Data: retained cohort-specific GWAS summary statistics for all age–trait analyses.
